# Development of a method for determining cheese-to-cheese stickiness in commercial cheese slice samples

**DOI:** 10.3168/jdsc.2025-0901

**Published:** 2026-02-06

**Authors:** Andrew Crumpley, Md Asaduzzaman, Hadi Eshpari, Prateek Sharma

**Affiliations:** 1 Department of Nutrition, Dietetics and Food Science, Utah State University, Logan, UT 84322; 2 Tillamook County Creamery Association, Portland, OR 97210; 3 Western Dairy Center, Department of Nutrition, Dietetics and Food Science, Utah State University, Logan, UT 84322

## Abstract

This study aimed to develop a practical tack testing method to measure the stickiness of commercial sliced cheese samples. When comparing instrumental variation, no significant differences were observed in tack force and tack energy measurement between the texture analyzer and the rheometer, confirming the suitability of the texture analyzer for routine measurement. A contact force of 10 N and contact time of 60 s were found suitable for measurement of cheese stickiness. The probe material surface types (aluminum vs. acrylic) did not show significant differences in terms of the tendency of the cheese to stick to the probe. A novel cheese-to-cheese setup for testing stickiness was developed which successfully differentiated between Cheddar and mozzarella cheese for their distinctive sticky behavior. This method presents a standardized approach for determining stickiness, which could enhance quality control and formulation strategies within the cheese industry.

## Highlights

• A novel method of testing cheese-cheese stickiness was developed on a texture analyzer.
• The method differentiated stickiness between Cheddar and mozzarella cheese slices.
• This method could be used to optimize cheese slice packaging and handling processes.

## Graphical Abstract

**Figure:**
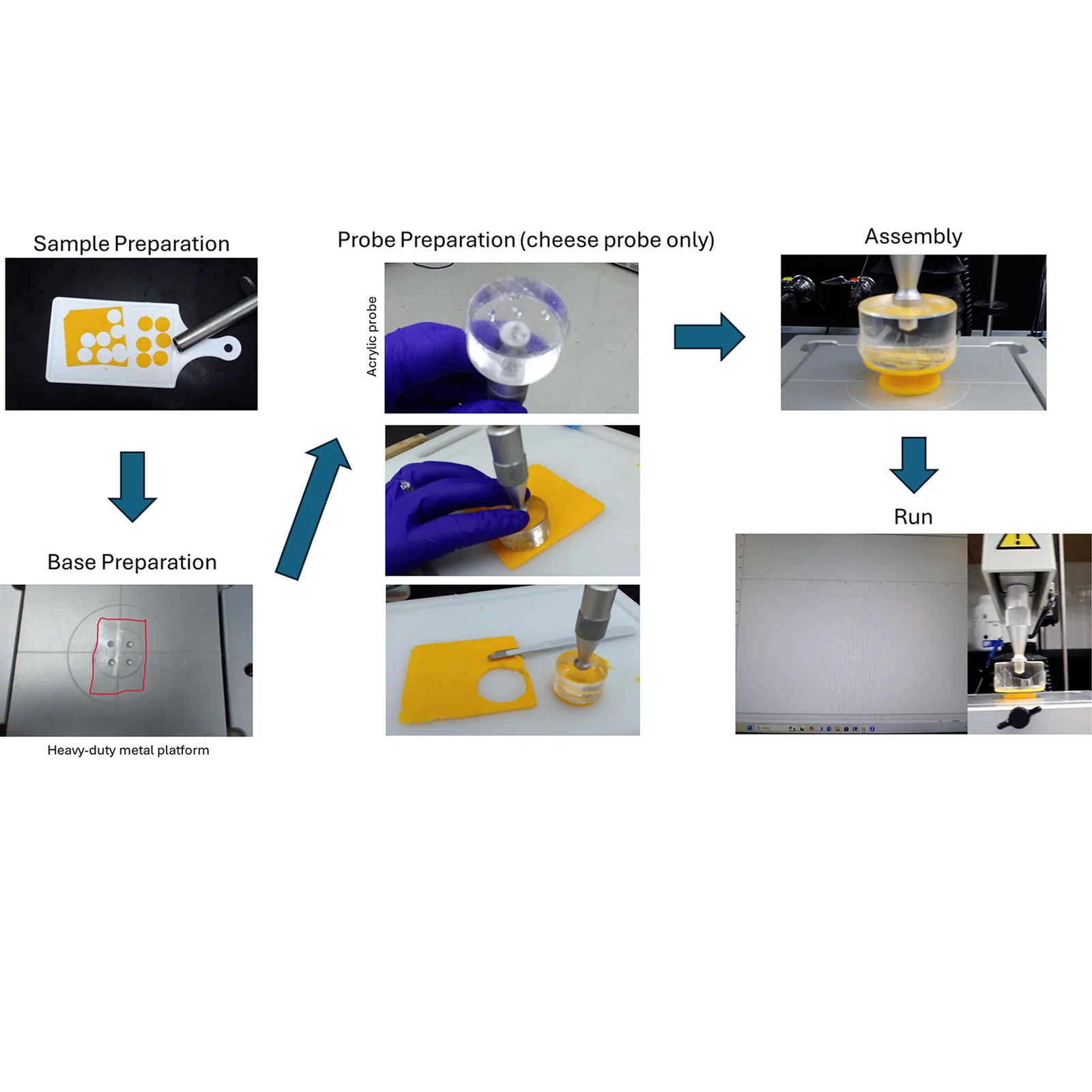
**Summary:** Commercially available cheese slices in retail format often present for consumers, such as an undesirable stickiness between 2 slices. This substantially undermines consumer convenience and may lead to the fragmentation of slices during handling and the end use, as separating the slices becomes more difficult. Despite the prevalence of this issue, the exact cause remains unclear. The initial step in elucidating the underlying causes of this issue involves quantifying the stickiness of cheese, with a particular focus on the adhesive forces present between 2 cheese slices. This article presents a novel method of testing cheese-cheese stickiness in commercial cheese slices. This method successfully differentiated between Cheddar and mozzarella cheese according to their distinctive sticky behavior. This method presents a standardized approach for determining stickiness, which could enhance quality control and formulation strategies within the cheese industry.

The cheese industry in the United States has demonstrated continued growth (production of 6.5 million metric tonnes in 2024), as evidenced by an increase in per-capita cheese consumption from 14.5 kg in 2000 to 19.1 kg in 2023, despite a concurrent rise (>90%) in cheese prices (Statista, 2023, 2025; Ibisworld, 2025). Sliced cheese represented a substantial portion of retail cheese sales, amounting to $2.4 million in 2022, thus ranking as the second most popular type of cheese format in terms of retail sales (Statista, 2024). Commercially sliced cheese offers advantages in terms of consumer convenience and consistency. It is pre-portioned into uniform slices, facilitating efficient preparation, ensuring standardized serving sizes, contributing to waste reduction, and providing economic benefits for both domestic and food service operations (Pace et al., 2024).

Cheese slicing operations sometimes encounter problems related to material loss due to either the crumbly nature of the cheese or due to a tendency to stick to moving metal surfaces (Sharma and Choudhary, 2025). On the other hand, commercially available cheese slices in retail format often present challenges at the consumer end, such as an undesirable stickiness between slices. This undermines consumer convenience and may lead to the fragmentation of slices during handling and the end use, as the challenge of separating them becomes more pronounced. Despite the prevalence of this issue, the exact cause remains unclear. However, there are some strategies used in the cheese industry to avoid the cheese-cheese stickiness problem and improve the consumer peeling experience, including the use of inner paper leaves with partial or full overlap or casting the molten process cheese mass into individually wrapped slices.

The initial step in elucidating the underlying causes of this issue involves accurately and objectively quantifying the stickiness of cheese, with a particular focus on the adhesive forces present between cheese slices. Adhesiveness in food samples is generally tested using texture profile analysis, which uses an empirical approach that is not very reliable or reproducible. Another method of testing is the descriptive sensory panel, which is subjective in nature. On the other hand, tack testing, a methodology employed in material science, evaluates tack force and tack energy, which are the forces and energies, respectively, that are necessary to cause separation between 2 adhesive surfaces. To perform a tack test, a circular metal plate is placed in contact with sample under a predetermined combination of contact time and force. Subsequently, the probe is retracted, and the resistance force exerted by the sample, which opposes the movement or separation of the probe, is measured and recorded. Prior research has applied tack testing as a measure of adhesiveness using a rheometer (Yildirim-Mavis et al., 2022) and texture analyzer (**TA**; Banville et al., 2013); however, it has predominantly focused on the tack forces between cheese and metal surface (Childs et al., 2007). To date, no method has been established to study the tack behavior between cheese-cheese surfaces (e.g., stickiness in cheese slices). Therefore, the objective of this research was to develop a user-friendly protocol for testing the stickiness between 2 slices. We hypothesized that such a protocol can be developed with the use of adhesive to attach cheese slices to both the top and bottom plates, through optimization of the contact force and duration and the separation speed.

A rheometer presents distinct advantages over a TA for determining the rheological properties of viscoelastic materials such as cheese because it provides precise control and measurement over stress and strain application, temperature, and separation speed. This precision enables highly reproducible and sensitive measurements, which facilitate a better understanding of the material properties of cheese (Biswas et al., 2008; Sharma, 2022). However, rheometers are expensive and require advanced training to operate, which limit their practical application in industrial quality control. Instead, a TA is faster and simpler, and is the universally used instrument for food texture analysis (Lis et al., 2021). Therefore, TA are preferred over rheometry for stickiness determination and are generally used at present in the cheese industry.

For developing a method for testing the stickiness of retail cheese slices, initial test parameters and techniques were based on the protocol described by Childs et al. (2007). Initial results obtained using this protocol showed high variability in measurements, prompting substantial adjustments to the test parameters. A variety of contact force and contact time combinations were tested to identify the optimum parameters to minimize variability and produce consistent data. A comparison of testing equipment (rheometer and TA) and probe types (stainless steel and acrylic) was also conducted to determine the suitability of the method for cheese-to-cheese testing. Finally, a methodological approach based on cheese-to-cheese contact in a TA was used to evaluate the differences in the stickiness of sliced Cheddar and mozzarella cheese.

Attempts were made to compare the cheese stickiness measurement using a modular compact rheometer (MCR302, Anton Paar USA Inc., Ashland, VA) using a PP50 probe made of stainless steel, as well as a Stable Micro Systems texture analyzer (TA.XT plus, Texture Technology Corp., Scarsdale, NY) using TA-30 (aluminum), TA-4 (acrylic), and TA-11ss (stainless steel) probes. Sliced mild Cheddar cheese (34.9% moisture, 26.0% protein, 33.0% fat, and 1.5% salt) and mozzarella cheese (45.6% moisture, 28.8% protein, 19.3% fat, and 1.23% salt) were purchased from the local supermarket (Walmart, Logan, UT) and stored at 4°C ± 1°C prior to use in the experiments. Cheddar and mozzarella cheeses were selected for the method development because they are expected to have perceivable differences in cheese slice stickiness due to their distinctive compositional and functional properties. A circular sample (7 cm diameter) was cut from a slice, sealed in a plastic bag, and stored refrigerated (4°C ± 1°C) until testing on the rheometer. Stainless steel probes were used on both instruments, and cheese samples were secured on the bottom geometry of both instruments. The applied force was set at 2.58 N for the rheometer and 10 N for the TA to ensure the same force per unit area of the cheese sample, allowing both instruments to apply ∼5,092 Pa to the sample. A force was applied for 60 s, and then the probe was removed from the cheese surface at a speed of 5 mm/s. Samples were measured in triplicate. In the case of the TA, tack force was equivalent to the maximum recorded value (Figure 1A), and for the rheometer, tack force was equivalent to the minimum recorded value (Figure 1B). Tack energy was the area under the curve for both instruments and was calculated in Microsoft Excel following the trapezoidal method (Sharma et al., 2016) using Equation [1]:

[1] $$tackenergy=\sum \frac{1}{2}\times \left(a+b\right)\times h,$$

where a and b are 2 parallel sides of the trapezoid and h is the perpendicular distance between parallel sides (Figure 1A, B). The results showed that there was no significant difference (*P* > 0.05) in tack force and tack energy values measured by the rheometer and the TA (Table 1), indicating the performance of the TA instrument for cheese stickiness measurement was at par with the rheometer for this testing. After this, we followed the protocol described by Childs et al. (2007) on a larger set of cheese samples, but we observed a large CV for our samples. Therefore, our next step was to determine the ideal test parameters using various combinations of forces (2–10 N) and times (5–60 s) to accurately and consistently measure the tack force and tack energy. In all cases, the probe was removed at a speed of 10 mm/s, 6 replications were run for each combination, and the CV for each force-time combination was evaluated.

**Figure 1:**
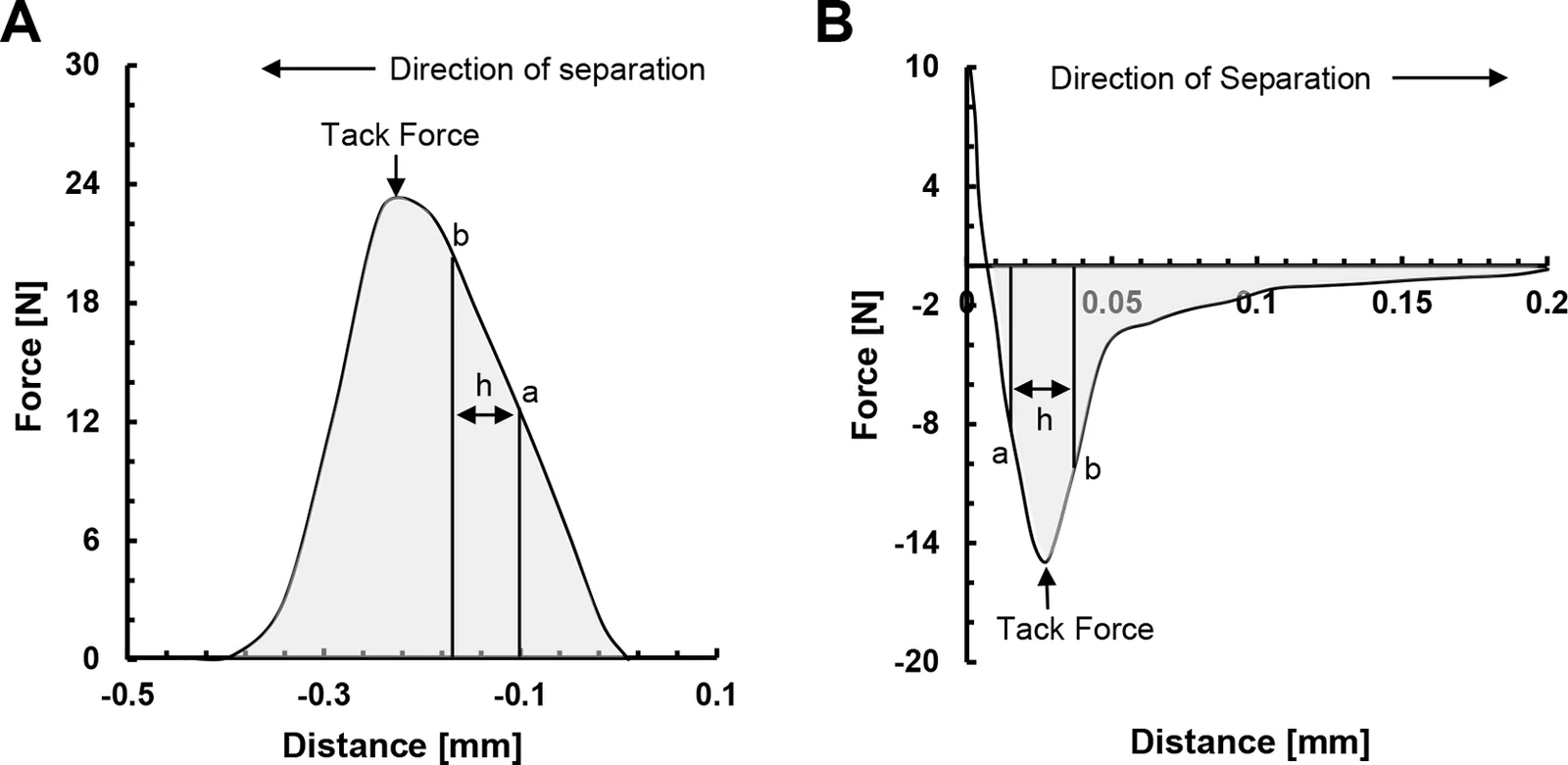
Deriving tack force and tack energy for the cheese-probe surface (SS) from the force-distance curve obtained from the texture analyzer (A) and rheometer (B). The highest and the lowest points of the curve indicate tack force. Tack energy is the area under the curve calculated by the trapezoid method (Sharma et al., 2016), where a and b are the force at a given distance and h is the distance.

**Table 1:** Tack force and energy of Cheddar cheese slices (cheese to probe surface) as measured at 4°C by rheometer and texture analyzer (for both instruments, contact force per unit area was kept the same); measurements were also conducted to optimize the contact force and contact time using the texture analyzer

Parameter	Tack force (N)	Tack energy (mJ)
Instrument^1^		
Rheometer	9.24 ± 2.11^a^	0.75 ± 0.23^a^
Texture analyzer	10.74 ± 1.13^a^	0.85 ± 0.11^a^
Contact time^2^ (s)		
5	8.92 ± 1.14^a^	1.10 ± 0.18^a^
15	13.54 ± 2.03^b^	2.07 ± 0.36^b^
30	18.87 ± 1.90^c^	3.05 ± 0.34^cd^
60	17.37 ± 1.35^c^	2.78 ± 0.31^bc^
Contact force^3^ (N)		
2	1.81 ± 0.70^a^	0.13 ± 0.05^a^
5	4.08 ± 1.26^b^	0.42 ± 0.14^b^
10	8.92 ± 1.14^c^	1.10 ± 0.18^c^

a–d Means with different superscripts within the same column are significantly different (*P* < 0.05) for rheometer and texture analyzer (n = 3).

1 Stainless steel probes were used on both instruments.

2 Contact force was 10 N with the acrylic probe for each contact time.

3 Contact time was 5 s with the acrylic probe for each contact force.

The results indicated that with increasing contact force from 2 N to 10 N (at contact time of 5 s), both the tack force and tack energy for cheese to probe surface (acrylic) increased significantly (*P* < 0.05; Table 1) and the CV reduced from 38.62% to 12.77% and 36.48% to 16.60%, respectively (Table 1 and Figure 2A). Similarly, with increasing contact time from 5 to 10 s (at contact force of 10 N), the tack force and tack energy increased significantly (*P* < 0.05) and the CV reduced from 12.76% to 7.79% and 16.60% to 11.14%, respectively (Table 1 and Figure 2A). Increasing contact time to ≥30 s resulted in the same CV for tack energy measurements; however, for tack force, the minimum CV was observed at 60 s. Therefore, a combination of a 10-N contact force and 60-s contact time was selected for further analysis, such as for cheese–probe surface and cheese-cheese contact. For comparing the effect of surface material of the probe, 1-inch (2.54 cm) circular samples were cut from the cheese slices using a circular borer and fixed on the TA base plate. Aluminum (TA-30) and acrylic (TA-4) probes were tested to measure the cheese stickiness, but no significant differences (*P* > 0.05) were observed between the aluminum and acrylic probes in measuring the tack force and tack energy between the probe and cheese surface (Figure 2B).

**Figure 2:**
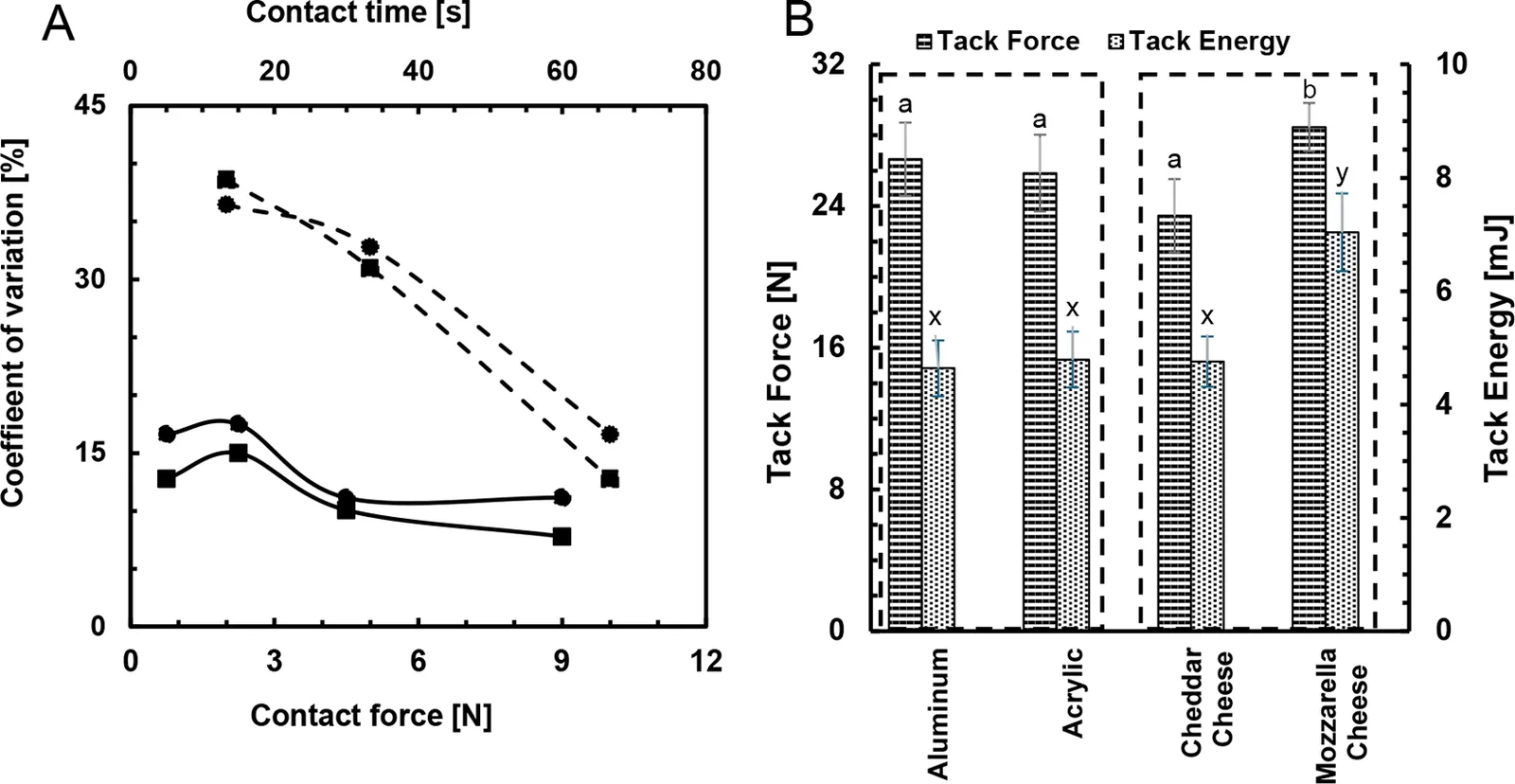
Effect of contact force (- -) and time (—) to minimize the coefficient of variation (n = 6) of tack force (▪) and tack energy (•) measurement (A) using an acrylic probe on the texture analyzer. Tack force and tack energy measured using different surfaces: Cheddar-aluminum, Cheddar-acrylic, Cheddar-Cheddar, and mozzarella-mozzarella (B). Different letters in the comparison of each parameter indicate a significant difference (*P* < 0.05; n = 3).

This was followed by the development of a new method to measure the stickiness parameter between cheese slices. The instrument probe material was made to adhere to the cheese surface, thereby replacing metal contact with cheese-to-cheese contact to mimic the commercial shelf-life conditions of sliced cheese. To attach the cheese sample to the TA-4 probe, the end of the probe was first covered entirely with permanent double-sided tape (Scotch, 3M Science, Maplewood, MN). Ethyl cyanoacrylate glue (Super Glue, Gorilla Glue Company, Cincinnati, OH) was applied to the tape, and the probe was pressed onto the cheese slice by hand for 30 s. Following this, extra cheese was trimmed from the probe, leaving the probe plane entirely covered by cheese. Another cheese slice was secured to a metal TA-90 base plate in a similar manner: first, the center of the base was covered with double-sided tape, then glue was applied to the tape, and finally, the cheese slice was gently pressed onto the base plate. While the glue was curing, the probe and base plate were covered with plastic wrap to prevent the sample from drying. Before applying a contact force, we ensured that the cheese samples were securely adhered to both the probe and base plate. Testing included the application of a 10 N force for 60 s, followed by the removal of the probe at a speed of 10 mm/s. The measurement was repeated 6 times. The results showed that the stickiness parameters, tack force, and tack energy were significantly different (*P* < 0.05) between Cheddar and mozzarella cheese (Figure 2B). This demonstrated that the new method can potentially be applied not only for measuring stickiness in sliced cheeses, but also to differentiate between different cheese types. This method can now be used to study the factors responsible for poor stickiness in retail cheese slices.

This study presents a reliable and reproducible protocol aimed at determining the stickiness of sliced cheese. Our results indicate that the use of a TA provides an effective means of measuring both tack force and tack energy as mean to evaluate stickiness, yielding results comparable to those obtained with standard rheometric methods. For the TA, an optimized setting of 10 N contact force and 60 s contact time resulted in significantly reduced measurement variability. Notably, the type of probe material (aluminum or acrylic) did not influence the results in terms of stickiness. Furthermore, a novel cheese-to-cheese fixture was developed for simulating commercial storage conditions, which differentiated the stickiness between Cheddar and mozzarella slices. These findings provide a standardized framework for food scientists and cheese industry professionals to assess and monitor cheese stickiness using a TA, thereby enhancing product development and quality control. Future investigations may apply this method to additional cheese varieties and explore its correlations with compositional and storage parameters.
